# Une cause rare d’hémorragie du post-partum: le thrombus génital

**DOI:** 10.11604/pamj.2019.33.241.18359

**Published:** 2019-07-19

**Authors:** Saad Benali, Jaouad Kouach

**Affiliations:** 1 Service de Gynécologie Obstétrique, Hôpital Militaire d'Instruction Mohamed V, Rabat, Maroc; 2 Faculté de Médecine et de Pharmacie de Rabat, Université Mohamed V, Rabat, Maroc

**Keywords:** Thrombus génital, post partum, hémorragie interne du post-partum, Genital thrombus, postpartum, internal postpartum hemorrhage

## Abstract

We report the case of a 30-year old primiparous patient, having given birth to a 3500g boy by cesarean section due to acute fetal distress. Two hours after childbirth, the patient had bilateral painful ecchymotic vulvar swelling suggesting post-partum thrombus of the vulva. Given the clinical stability of the size of this hematoma, the absence of unbearable pain and the stable hemodynamic status, conservative medical treatment (ice bag + NSAIDs). Output was favorable with progressive decrease in the size of the hematoma until disappearance 8 weeks after delivery. Thrombus or genital hematoma is a rare but potentially very serious post-partum haemorrhagic complication. There are four major types of genital hematomas: hematoma of the vulva, vulvo-vaginal hematoma, vaginal hematoma and pelvic-abdominal hematoma. The contributing factors are primiparity, instrumental extractions, toxaemia of pregnancy, twin pregnancies and vulvovaginal varicose veins. It is essential to suspect it in patients with pain and/or internal postpartum hemorrhage, and, if any doubt exists, to perform vulvovaginal inspection. Early treatment is necessary and should be associated with hemodynamic resuscitation, first-line surgery and percutaneous radiologic arterial embolization in case of failure of surgery.

## Image en médecine

Patiente âgée de 30 ans, primipare, ayant accouché par césarienne pour souffrance fœtale aigüe, d'un garçon de 3500g. A deux heures du post-partum, la patiente a installé une tuméfaction ecchymotique vulvaire bilatérale douloureuse en faveur d'un thrombus vulvaire du post-partum. Devant la stabilité de la taille de cet hématome à la surveillance, l'absence de douleur insupportable et l'état hémodynamique stable, on a opté pour un traitement médical conservateur (vessie de glace + AINS). L'évolution était favorable avec une diminution progressive de la taille de l'hématome jusqu'à disparition à 8 semaines du post-partum. Le thrombus ou hématome génital est une complication hémorragique rare du post-partum, mais potentiellement gravissime. On distingue quatre grandes sortes d'hématomes génitaux: l'hématome vulvaire, l'hématome vulvo-vaginal, l'hématome vaginal proprement dit et l'hématome pelvi-abdominal. Les facteurs favorisants sont la primiparité, les extractions instrumentales, la toxémie gravidique, les grossesses gémellaires et les varices vulvovaginales. Il faut savoir l'évoquer devant toute symptomatologie douloureuse et/ou d'hémorragie interne du post-partum et réaliser au moindre doute une inspection vulvovaginale. Le traitement doit être appliqué rapidement et associe à la réanimation hémodynamique, la chirurgie en première intention et l'embolisation artérielle radiologique percutanée en cas d'échec de celle-ci.

**Figure 1:**
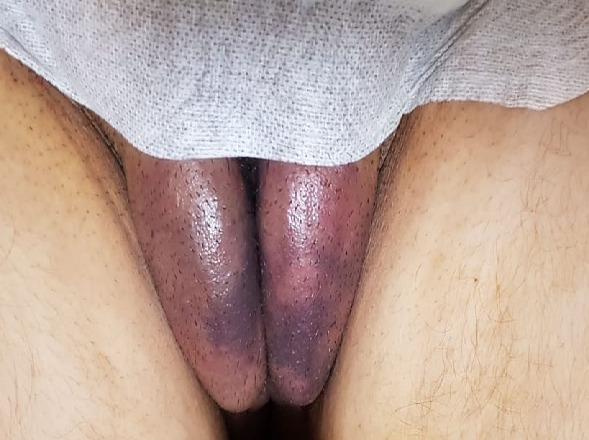
Le thrombus génital

